# Mr. Edwards's Case of Ulcerated Cornea

**Published:** 1810-01

**Authors:** G. F. Edwards

**Affiliations:** Member of the Royal College of Surgeons, London. Bath


### *To the Editors of the Medical and Physical Journal.*

Gentlemen,

A Singular morbid appearance of the crystaline cornea falling under my care, induced me to make a drawing, which delineates the disease as accurately as the inflamed state of the eye would permit; shewing that it is possible, in a short time, for that part of the organ to be destroyed by ulceration,—I have herewith transmitted the same, with the history of the case, for insertion in your valuable Journal.

Mrs. F. whose occupation is that of nursing females after their accouchement, was seized with sensations like dust or other extraneous substances in her left eye, which were soon followed by considerable pain, heat, and inflammation, with a discharge of matter, but in no material quantity. These symptoms increased, till at length the eye became so painful, as to oblige her to quit the situation she then held, and return home. At the end of five days from the commencement of the attack, she experienced darting pains through the head and globe of the eye, with swelling, tension, and a sense of enlarged feel.

Every

Every thing prescribed by her friends and neighbours was tried without success, which determined her to get other advice. Unfortunately, she applied to a person whose knowledge of the eye and whose medical skill were not any ways superior to those already consulted. A quantity of medicines had been taken, but no remedy applied to the affected part, consequently the disease made rapid strides, and proved to this poor woman that she had been trifled with. As she found something escape from her eye like jelly, it alarmed her, and she applied to me.

On examination, the first object which caught my attention was the iris protruding through an aperture in the crystaline cornea, evidently caused by ulceration, produced in all probability by continued and neglected inflammation. The tunica sclerotica and conjunctiva were highly inflamed; and by reference to the drawing, blood-vessels may be seen running into the cornea, a triangular portion of which was quite opake, and bore marks of ulceration through its whole substance. The pupil was contracted towards this opacity, and was scarcely sensible to the glare of a strong light. The remaining portion of the cornea was in a state of inflammation, of a red brown colour. The aqueous humour had been discharged some days previous to the vitreous, evidently proving that as the ulceration proceeded, the opening in the cornea became larger; so large, as to permit the escape of a part of the vitreous humour; but I could not ascertain that the crystaline had been forced out by the action of the muscles, because if this had been the case, my patient could not have discovered light, and the membranes would have sunk to the bottom of the orbit, and no art could have restored them; but this glare was only such in proportion to the quantity of humours that had escaped; and I am of opinion, that only a small part of the vitreous humour had been forced out.

In this state I found the poor woman, enduring excruciating pains in her head, violent inflammation of the coat of the eye, loss of sleep, great heat and thirst, accompanied with a white dry tongue, and pulse 120 in a minute. Leeches were applied to the temple, and a few ounces of blood taken from the arm; warm fomentations of decoct. pap. alb. constantly applied; a cathartic purge, and small doses of opium after the bowels were evacuated. Some of the large turgid blood vessels I divided with scissars; and in two days this treatment was the means of giving very considerable relief; the inflammation abated, the discharge lessened, the pain in a great measure ceased, sleep returned, and fever diminished. The same plan was persisted in, and a collyrium, composed of Aq. ammon. a. ʒij. aq. rosæ ℥jß. extr. Saturni gtt. ij. tinct. opii vin. gtt. xv. was applied to the inflamed surface by means of a tea spoon. This collyrium induced the vessels to contract to their natural size, and by its anodyne and cooling properties gave very considerable relief, so much so, that she wished to apply it oftener than necessary. In a few days the above was changed for the subsequent. ℞. Aq. zinci. vit. e camph. ʒij. aq. flor. samb. ℥ij. misce; and by steadily pursuing it with all the other plans of depletion, I am happy to say that my patient has regained more than reasonably might have been expected.

I am aware that it may be advanced by some, that the cornea burst from over distention, effected by effused matter within the cavity of the eye, or by some other disease, such as hydrophthalmia, staphyloma, &c. This I shall deny as being the fact, because evident marks of ulceration acting externally are now visible; and for this obvious reason, viz. that the aqueous humour escaped some days previous to the vitreous, through one or more perforations made by the matter of ulcer. It appears from the whole, that inflammation was the first cause, which was rapidly succeeded by ulceration and destruction of a portion of the cornea. The inflammatory action effected, no doubt, the whole of the membranes, and destroyed the sensibility of the retina, which was one cause of the contraction of the pupil.*

I am, &c.

G. F. EDWARDS,

Member of the Royal College of Surgeons, London.

*Bath, Dec. 12, 1809.*

---

*Account of a diseased Testicle, successfully removed at an advanced Stage*; by Mr. JOHN TAUNTON, Surgeon to the City and Finsbury Dispensaries, &c.

S. S. æt. 35, applied to me for advice June 9, 1809. He had perceived a small swelling at the bottom of the scrotum about twenty months previous to my seeing him; this

---

* A coloured engraving of this case will be given in a future Number of this Volume.

